# Supplementary material for: MaCts1, an Endochitinase, Is Involved in Conidial Germination, Conidial Yield, Stress Tolerances and Microcycle Conidiation in Metarhizium acridum
Source: Biology (Basel). 2022 Nov 29;11(12):1730. doi: 10.3390/biology11121730 (PMC9774441; doi:10.3390/biology11121730)
Supplement: Supplementary file 1 [file biology-11-01730-s001.zip › biology-2027680-supplementary.pdf]

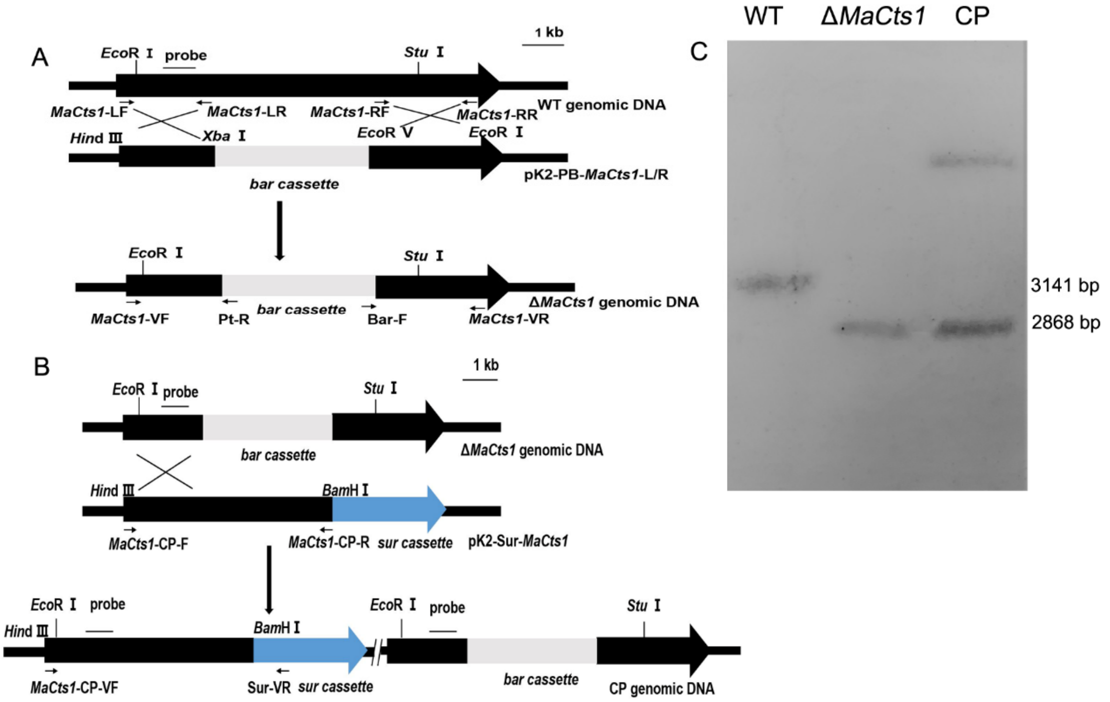

Figure S1. The disruption and complementation of *MaCts1*. (A) *MaCts1* disruption vector was constructed based on pk2-PB vector. (B) *MaCts1* complementation vector was constructed based on pk2-Sur vector. (C) The WT, Δ*MaCts1* and CP were verified by Southern blotting. The genomic DNA was cut with *Eco*RI and *Stu*I. The Probe was amplified from genomic DNA by PCR using primer pair *MaCts1*-TF and *MaCts1*-TR (Table S1).

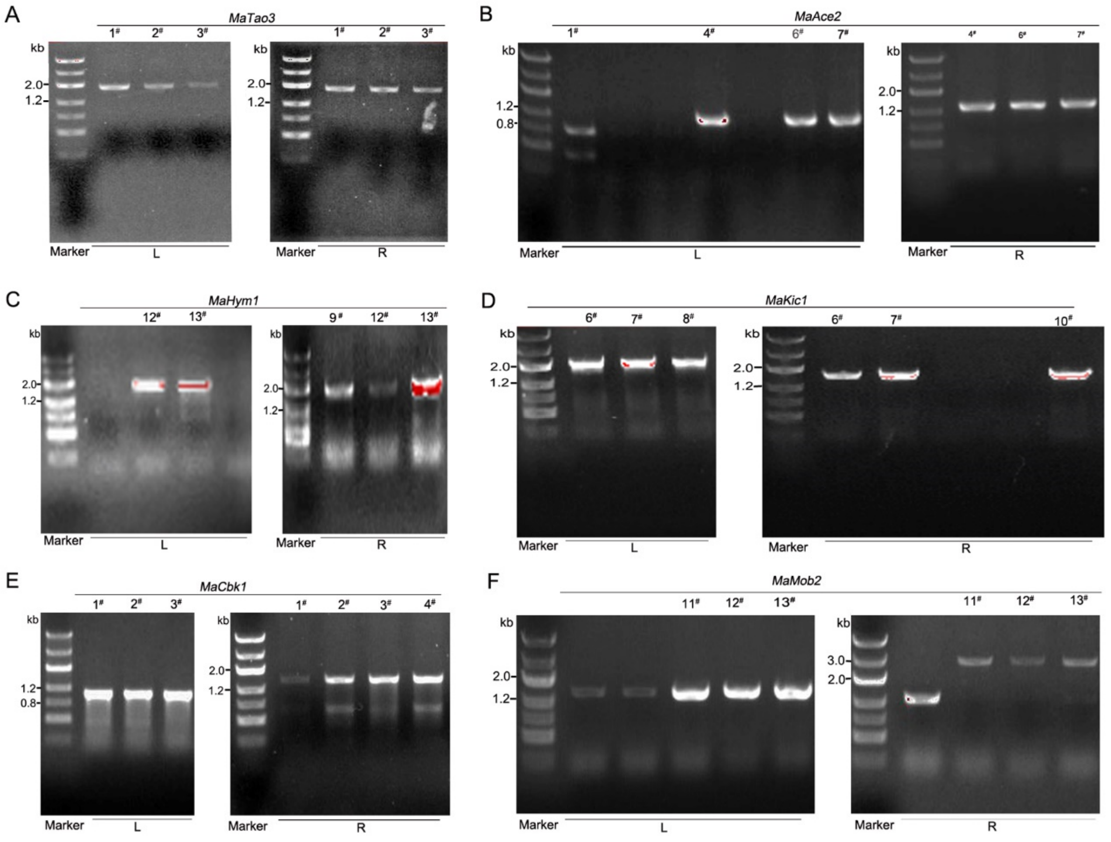

Figure S2. Disruption of key genes, including *MaHym1*, *MaKic1*, *MaTao3*, *MaCbk1* and *MaMob2*, in MOR pathway and *MaAce2* in *M. acridum*. All mutants were verified by PCR analyses. All primers used in this study were listed in Table S1.

Table S1. Primers used in this work

Primers	Sequence (5′-3′)	Remarks
MaCts1-LF	GTACGCAATCAGTGAGGGGT	Used to clone the 5′ end of *MaCts1*
MaCts1-LR	TTGAAGCGGTCTAATACCAC	
MaCts1-RF	GCAGGGGTATGAAATTGTGT	Used to clone the 3′ end of *MaCts1*
MaCts1-RR	TAAGGTAGCAGGAGATAGTG	
MaCts1-VF	CCTTTCCCTCGGTCCATTAT	Used for screening the *MaCts1*-disruption transformants
MaCts1-VR	CCGAAGCATCTAAGACTAAG	
Pt-R	CAGCCAAGCCCAAAAAGTG	
Bar-F	GCTCTACACCCACCTGCT	
MaCts1-T-F	AGGTAACTTGATGGGCACAC	Used to clone the probe of *MaCts1*
MaCts1-T-R	GAAGATTGACTCGTTGACCG	
MaCts1-CP-F	AACAGGGCAATAGGAGAAAG	Used to form pK2-*MaCts1*-EGFP-SUR vector
MaCts1-CP-R	TTGGCAGGAAGACCACCATT	
EGFP-VR	CGATGCGGTTCACCAGGGTGT	Used for screening the *MaCts1*-complement transformants
MaHym1-LF	TCAACCAAGGGGAAAGACAC	Used to clone the 5′ end of *MaHym1*
MaHym1-LR	ACGACATCAATACTTCCGCC	
MaHym1-RF	GAGGGCTATTTCGGTTTGAG	Used to clone the 3′ end of *MaHym1*
MaHym1-RR	GGAAAGACGACATCAAATGC	
MaHym1-VF	TACCCTTCACTTTACCTACC	Used for screening the *MaHym1*-disruption transformants
MaHym1-VR	TGATTCCATACCTTGTGTCC	
MaKic1-LF	CAAGCAGGAGCAAAACAGAG	Used to clone the 5′ end of *MaKic1*
MaKic1-LR	CCAATGCTGTGCCTGTAGAC	
MaKic1-RF	CGGCAATGGAGGATGAAATG	Used to clone the 3′ end of *MaKic1*
MaKic1-RR	CCCCTCGTTCCTTTTCTTGT	
MaKic1-VF	GATAATGGTCTATGCGAGGC	Used for screening the *MaKic1*-disruption transformants
MaKic1-VR	ACAGGCGTAGTTTTGAGATG	
MaTao3-LF	GACTGGCATCAACACATCAG	Used to clone the 5′ end of *MaTao3*
MaTao3-LR	AGCTAAAAGGGCATGGCAGT	
MaTao3-RF	ATACAAATCTACGCAGGGAG	Used to clone the 3′ end of *MaTao3*
MaTao3-RR	CGCAAACCAACCGCCATCAT	
MaTao3-VF	AGGCACGGATACTCAAGCAT	Used for screening the *MaTao3*-disruption transformants
MaTao3-VR	GTCCTCTTCACCACCATCTT	
MaMob2-LF	ACTATTATGAACCGGCAGCCATCC	Used to clone the 5′ end of *MaMob2*

MaMob2-LR	ACCGTCGGCGTGAGGCAAGA	
MaMob2-RF	TCTGAGTTTGAGGGCGTGTC	Used to clone the 3′ end of *MaMob2*
MaMob2-RR	AGTCCAAGCGGTGGCGATG	
MaMob2-VF	GAAGTTTGTGGTTCGCAGTC	Used for screening the *MaMob2*-disruption transformants
MaMob2-VR	TGAGCACGGCATCAATACA	
MaCbk1-LF	GCTCAGGACAGGGTTCGTTC	Used to clone the 5′ end of *MaCbk1*
MaCbk1-LR	CCTCGTGTTGCTGCTTCTTT	
MaCbk1-RF	TGGCGCAATATGTTCAATG	Used to clone the 3′ end of *MaCbk1*
MaCbk1-RR	GGCAAGAAGCACCAGACC	
MaCbk1-VF	AAAGCCCGTGATGTCTGC	Used for screening the *MaCbk1*-disruption transformants
MaCbk1-VR	CACTCCGCCACCAACG	
MaAce2-LF	CGAGACTCCCGACTTGACC	Used to clone the 5′ end of *MaAce2*
MaAce2-LR	TGCTTGTTCCCGATTTAGC	
MaAce2-RF	GGAATGCTTTCTGCGACAA	Used to clone the 3′ end of *MaAce2*
MaAce2-RR	CACACCGCTTCACCTTTCAT	
MaAce2-VF	ACGCTCCCGAAACGACC	Used for screening the *MaAce2*-disruption transformants
MaAce2-VR	AGACGAAATGCTCACGCC	

Table S2. Identification of RAM/MOR components in *M. acridum*

Species	*S. cerevisiae*		*S. pombe*		*U. maydis*		*M. acridum*
	homologue	E value	homologue	E value	homologue	E value	homologue
RAM/MOR components	Hym1	$4e^{-37}$	Pmo25	$1e^{-75}$	Hym1	$1e^{-75}$	Hym1
	Kic1	$5e^{-80}$	Nak1	$7e^{-101}$	Don3	$5e^{-125}$	Kic1
	Tao3	0.0	Mor2	$6e^{-168}$	Tao3	0.0	Tao3
	Cbk1	0.0	Orb6	0.0	Ukc1	0.0	Cbk1
	Mob2	$4e^{-33}$	Mob2	$4e^{-39}$	Mob2	$5e^{-28}$	Mob2
	Ace2	$4e^{-26}$					Ace2

The homologous protein names and E values of all components are reported in a separate column for each specie.
